# Variable feelings of cohesion, trust, individualism and exclusion and their consequences on Swiss public mental health during the ongoing COVID-19 pandemic

**DOI:** 10.1192/j.eurpsy.2022.1375

**Published:** 2022-09-01

**Authors:** F. Kraxner

**Affiliations:** Spital Affoltern, Psychiatry And Psychotherapy, Affoltern am Albis, Switzerland

**Keywords:** Covid-19, Switzerland, public mental health, case description

## Abstract

**Introduction:**

The first cases of COVID-19 in Switzerland were related to the Milan cluster in February 2020. Border crossing restrictions were imposed and economic support measures worth 40 billion Swiss francs were announced. By 24th September 2021 Switzerland achieved a fully vaccination rate of 54%, confirmed 836’000 cases and 11’060 deaths.

**Objectives:**

The objective was to describe and analyse the mental health of the general Swiss population under the ongoing COVID-19 pandemic and it’s social changes: including a case description, transgenerational influence and psychosocial treatment opportunities

**Methods:**

To answer the research question, I used deepened internet research, population interviews among different age gropus and colloquies with healthcare providers and federal authorities.

**Results:**

Different psychosocial phenomena lead to the transgenerational influence. Unfortunately, vaccination rate is slowed down by this pandemic’s impacts on public mental health. While during the first wave the positive feelings of cohesion and trust dominated, the mood changed afterwards to anxiousness and reactive individualism. Among all generations the fear of infection was shown to be a general booster of anxiety and distress. In fact, Swiss experts perceive the corona crisis as a catalysator for depression and anxiety disorders.

**Conclusions:**

Specific psychosocial treatment has to address general and individual vulnerability factors. However, staying in touch with family members, pursuing meaningful activities and being physically active can all help to overcome COVID-19 related mental health issues.

**Disclosure:**

No significant relationships.

